# Predictors of exclusive breastfeeding practice among nursing mothers attending a health care facility in a peri-urban setting in Lagos State, Nigeria

**DOI:** 10.4314/ahs.v22i2.63

**Published:** 2022-06

**Authors:** Micheal Ayodeji Sokan-Adeaga, Adewale Allen Sokan-Adeaga, Eniola Deborah Sokan-Adeaga, Akin Osibogun, Hoseinzadeh Edris

**Affiliations:** 1 Department of Community Health and Primary Health Care, Faculty of Clinical Sciences, College of Medicine, University of Lagos, Lagos, Nigeria; 2 Department of Environmental Health Sciences, Faculty of Public Health, College of Medicine, Lead City University, Ibadan, Nigeria; 3 Department of Environmental Health Sciences, Faculty of Public Health, College of Medicine, University of Ibadan, Ibadan, Nigeria; 4 Department of Physiology, Faculty of Basic Medical Sciences, College of Medicine, LadokeAkintola University of Technology (LAUTECH), Ogbomosho, Oyo State, Nigeria; 5 Incubation and Innovation Center, Saveh University of Medical Sciences, Saveh, Iran

**Keywords:** Exclusive breastfeeding practice, Nursing mothers, Predictors, Infant feeding, Child index

## Abstract

**Background:**

The Nigerian government initiated various national infant and young child feeding programmes (1992–2005) to improve juveniles well-being. Despite these efforts, under-five children feeding related diseases and mortality still escalate. Investigating the drivers of exclusive breastfeeding (EBF) is exigent in tackling this menace.

**Objective:**

To investigate maternal socio-demographic and index child variables that serve as predictors of EBF practice among nursing mother attending a healthcare facility in Lagos, western Nigeria.

**Methods:**

One hundred and twenty (N=120) consenting nursing mothers (15–49 years) with infants between 0–24 months completed a structured, self-administered questionnaire. Scores of current practice level for EBF was computed and adjusted odd ratios (aORs) generated from a logistic regression model.

**Results:**

Respondents mean age was 28.7 ± 2.3 years. Of 120 respondents, 82(68.3%) and 38(31.7%) had good and poor EBF practice respectively. Having an index child <6months age (aOR=5.02, 95% confidence intervalCI=1.28–15.43), being in monogamy (aOR=3.0, 95% CI=1.80–6.73), having tertiary education (aOR=3.12, 95% CI=1.39–8.96), being married (aOR=2.0, 95% CI=0.1–0.8) and vaginal delivery (aOR=2.96, 95% CI=1.75–7.48) increased the odds of EBF practice.

**Conclusion:**

Age of index child, marriage type, maternal education, marital status and nature of delivery independently predicted EBF practice.

## Introduction

Poor breastfeeding, mostly non-exclusive breastfeeding in the first semester of life, is estimated to have led to 1.4 million mortality and 10% of diseases among under-fives and disability of 44 million worldwide. It is also associated with chronic effects such as ill academic result, diminished efficiency, and retrograde cognition and sociality^1^–^3^. Consequently, this has led to international bodies such as the World Health Organization (WHO) and United Nations Children's Fund (UNICEF) to issued new ten-step guidance on April 11, 2018 in Geneva to corroborate support for breastfeeding in health facilities that offer maternity and newborn services. These ten steps to successful breastfeeding underpin the Innocenti Declaration (1990) and the Baby-Friendly Hospital Initiative (1991) launched by both organisations^4^. This practical guidance urges young mothers to breastfeed and informs health workers on best pratices that aggrandise breastfeeding. Therefore, it has been recommended by WHO and UNICEF that all women should breastfeed their babies exclusively in the first semester and thereafter with supplementary feeding and continue breastfeeding up to 24 months or beyond for wholesome growth and development; support mothers to instigate and sustain breastfeeding and manage frequent challenges; facilitate prompt and uninterrupted skin-to-skin contact and support mothers to initiate breastfeeding immediately after after birth; and breastfed neophytes only with breast milk without any food or fluids unless medically recommend^5^–^6^,^4^.

In a bid to create awareness and to accentuate breastfeeding practices for salubrious and wholesome impacts on infants, young children and mothers. The Nigeria's government in an effort to attain the targets of the 1990 Innocenti Declaration launched a National Guidelines on the Baby Friendly Hospital Initiative (BFHI) in 1992, the National Breastfeeding Policy in 1998, the National Policy on Food and Nutrition in 2001 and the National Policy on Infant and Young Child Feeding (IYCF) in Nigeria in 2005, to provide guidance on multiple issues associated with the promotion of breastfeeding and complementary feeding to national and local tiers of government and to health practitioners and other health stakeholders. Also, the Federal Ministry of Health emphasises early initiation of breastfeeding within one hour of birth, exclusive breastfeeding (EBF) for the first semester of life, continued breastfeeding for two years or beyond, with the introduction of appropriate complementary foods from six months. However, these programmes have not translated to giant stride in the national implementation of exclusive breastfeeding practice owning to the fact that most policy documents were available only at the national level; while at the state level and below, nutrition-related policies, reports, and tools were rarely available, even within the State Ministry of Health (SMOH).

Although exclusive breastfeeding is associated with numerous benefits which serve as adequate incentives for mothers to practice it, yet, several mothers were indispose or apathetic to exclusively breastfeed their infants^7^. According to the National Demographic and Health Survey (NDHS) 2018, the early initiation rate of breastfeeding is 42% which obviously depicts that less than 50% of the neonates in the country are breastfed within one hour of birth. Also, the exclusive breastfeeding rate in Nigeria is estimated at 29%, which is a pointer to the fact that only a small percentage of infants aged 0–6 months are exclusively breastfed leaving a whopping 71% of babies not enjoying the benefits of breast milk in their formative years. Only 9% of organizations have a workplace breastfeeding policy^8^. Agunbiade and Ogunleye^9^ reported that some nursing mothers in Nigeria deter EBF practice due to the misconception that their breasts will sag when they exclusively breastfeed their babies, thus rendering them sexually unappealing to their their spouses. Also, Aborigo et al.^10^ enunciated that in Ghana, younger mothers did not practice EBF for reason of not losing their breast shape. Kakute., et al.^11^ reported that mothers from some ethinic groups in rural Cameroon introduce water or food prior to six months of age, with about 40% introducing water to babies in the first month of life. In Ethiopia, 49% of infants were exclusively breastfed in the first semester of life, while 56.9% were exclusively breastfed for the first four months^12^–^14^. Moreover, several researchers in western African had reported that the practice of giving water to neonate to quench their thirst is also an impedeing factors to EBF practice by mothers^15^–^19^,^11^,^20^.

Several studies have considered impact of maternal demographics, cultural beliefs and health care system on breastfeeding practice^21^–^23^. Howbeit, these factors are still vaguely defined. Furthermore, there is an apparent dearth of empirical data on predictors of EBF practice among nursing mothers in sub-Sahara Africa (SSA) having infants between 0 – 24 months of age. This study is a timely intervention as it helps to fill the knowledge gaps by identifying the socio-demographic variables and child index factors influencing exclusive breastfeeding practice among nursing mother. By and large, findings from this study will serve as an advocacy tool for policy makers and public health practitioners to promote EBF campaign among nursing mothers in Nigeria and other sub-Saharan countries. Hence, this study determine the predictors of exclusive breastfeeding practice among nursing mothers attending a health care facility in a peri-urban setting in Lagos State, Nigeria.

## Methods

### Study Design and Settings

We conducted a cross-sectional survey among nursing mothers attending the IsheriOlofin Primary Health Centre (IOPHC) situated in Egbe/Idimu Local Council Development Area (LCDA) in Alimosho Local Government Area (LGA) of Lagos State, Nigeria. Lagos State famously known as the “Centre of excellence” was established in May 27 1967. It is situated in the South Western part Nigeria and bordered by Ogun State both in the North and East, and Republic of Benin on the West. In the South, it stretches for 180 km along the coast of the Atlantic Ocean and occupies an estimated area of 3,577sq km, of which 22% or 787sq km consists of lagoons and creeks. It serve as a commercial hub for all Nigerians and it is domiciled mostly by Nigerians of divergent cultures and origins^24^. The state is divided into twenty (20) administrative units called local government areas (LGAs).

### Study Population

The study population consists of mothers aged 15 to 49 years who are currently breastfeeding infants between 0 to 24 months and who are attending postnatal clinic, immunization unit, family planning unit, special care baby unit, children out-patient clinic at IOPHC.

### Sample Size

A total of one hundred and eighty-seven (187) registered nursing mothers were attending the IOPHC as at the period of the study. From this sample frame, one hundred and thirty-three (133) of the nursing mothers consented and participated in the study, but only 120 of the participants completed and correctly filled the questionnaires.

### Sampling Technique

A non-probability sample technique was adopted in the selection of participants for the study. At each clinic visit during the period of the study, eligible participants who shown willingness and gave verbal consent to participate in the study were enrolled.

### Definition of Variables

The following operational terms were adopted in the study vis:

- Exclusive Breastfeeding: not giving any other food or drink, not even water, except breast milk (including milk, expressed or from a wet nurse) for six (6) months of life, but allows the infant to receive Oral Rehydration Solutions (ORS), drops and syrups (vitamins, minerals and medicines).
- Good Practice: practice that is not only good, but has been proven to work well and yield good outcomes, and is therefore suggested as a model.
- Poor Practice: practice that fail to provide a good standard of care, support or outcome; and if allow to continue can cause harm and can become abuse.
- Predictors: variables that are linked with particular outcomes. They are extensions of correlational statistics.

### Data Measures

We are oblivious of any previous questionnaires specific to predictors of current infant feeding practice among nursing mothers visiting a health facility in a peri-urban setting hence we formulated questions to elicits information on socio-demographic variables of respondents, respondent child's vital information and current infant feeding practice among respondents.

### Instrument for Data Collection

Data collection was done through a pre-tested structured, self-administered questionnaire. The questionnaires were designed by the authors. The components of the questionnaire were categorized into three (3) sections namely: Section A: socio-demographic information of respondents; Section B: respondent child's vital information; and Section C: respondents Infant Feeding Practice.

- Socio demographic characteristics: this section included variables such as age at last birth day, sex, ethnic group, educational status, marital status, type of marriage, religion etc.
- Childs vital information: Child's age, birth order, child sex, nature/type of delivery.
- Exclusive Breastfeeding/current infant feeding practices of mothers: This is to determine if Exclusive Breast feeding is practiced and if not why. EBF practice was assessed using 6 items with correct answers attracting 2 marks each, the overall total was 12 marks, and the mean score was 8 marks. Less than 8 marks was indicated as poor practice while 8 marks and above was indicated as good practice. The scoring and pass mark was arbitrarily allocated.

### Validity and Reliability of the Instrument

The instrument was pre-tested amidst nursing mothers visiting Egan Primary Health Centre (EPHC), Egan, Igando/Ikotun LCDA, Lagos, Nigeria. During the pretest, the questionnaire was administered only to consenting individuals present at facility. The Cronbach's Alpha method was used to determine the instrument reliability. The alpha-coefficient for the pre-test was 0.78, which was an indication of the reliability of the questionnaire.

### Data Collection Procedure

The data was collected over a period of ten (10) weeks, and it was done either by face-to-face interview or through self-administration of the questionnaires. To ensure that we collect accurate information, the self-administered format was only permitted for nursing mothers with tertiary education and who indicated willingness to do so.

The consenting nursing mothers were recruited into the study after thorough explanation of the research. Participants were informed of their right to voluntary withdrawal from the research at any instance, if they desire to discontinue without any penalty; and that there was no benefit attached to participation except for findings obtained from the study.

### Data Management and analysis

Data was entered and analysed using SPSS Package version 24 (SPSS, Inc.; USA). The use of frequency tables, percentages, chart presentation and descriptive statistics (mean and standard deviation) were utilised for result presentation.

Inferential statistics such as Chi-square (χ2) was employed in testing the association between the independent variables (socio-demographic variables and respondents' index child) and outcome variable (exclusive breastfeeding practice). Also multiple logistic regressions model was used to assess how each predictor (demographic factors and child's vital information) is likely to influence and contribute to the outcome (infant feeding practices). Statistical significance was place at one-tailed, p-value less than 0.05 for all inferential analysis.

### Ethical Considerations

An introductory letter was obtained from the Medical Officer of Health (MOH) at the Egbe/Idimu LCDA after studying the research proposal. This was subsequently approved by the management of the IOPHC. Also, informed verbal consent was obtained from all participants after thorough explanation of the research before recruited into the study. The right to privacy and anonymity of the participants in the study were strictly adhered to by the researchers and respondents were assured that information provided would be used for research only.

## Results

A total of 133 questionnaires were administered and of which only 120 were completed, returned and subsequently analysed giving a response rate of 90%.

### Socio-demographic Characteristics of Nursing Mothers

The baseline socio-demographic characteristics of respondents are shown in Table 1. The mean respondents' age was 28.7 ± 2.3years. Almost (99.2%) of the respondents were married and majority (92.5%) was in a monogamous marriage. Most (80.8%) of the respondents were Christians and majority (52.5%) was of the Yoruba ethnic group. Most (52.5%) of the respondents had tertiary education (52.5%) while a few (7.5%) had postgraduate education. Most (90%) of the respondents resides in the urban area. The highest proportion (36.7%) of the respondents were traders and nearly one-third (31.7%) of the nursing mothers had two (2) living children.

**Table 1:** Socio-Demographic Characteristics of Respondents

Variables	Frequency (N=120)	Percentage (%)
**Age group (year)**		
≤29	62	51.7
30–34	39	32.5
≥35	19	15.8

**Marital Status**		
Married	119	99.2
Divorced/Widow/Separated/Co-habiting	1	0.8

**Types of Marriage**		
Monogamous	111	92.5
Polygamous	9	7.5

**Religion**		
Christianity	97	80.8
Islam	22	18.3
Traditional worshipper	1	0.8

**Ethnicity**		
Yoruba	63	52.5
Igbo	29	24.2
Hausa	6	5.0
Others	22	18.3

**Educational Status**		
Primary	4	3.3
Secondary	44	36.7
Tertiary	63	52.5
Postgraduate	9	7.5

**Residence**		
Rural	12	10
Urban	108	90

**Occupation**		
Full-time house wife	16	13.3
Trader	44	36.7
Artisan	19	15.8
Civil servant	41	34.2

**Number of living children**		
1	37	30.8
2	38	31.7
3	31	25.8
≥4	14	11.7

### Information on Respondents' Index Child Attending IsheriOlofin Primary Health Centre (IOPHC), Lagos

Table 2, shows that more than half (60.8%) of the respondents index child were less than 6 months of age with mean age of 90.4±7.1 days. There was a higher proportion of males children (52.5%) than female (47.5%); and 35.0% of the children were in the second position (birth order) from the same mother. Most (84.2%) of the respondents' had their child through normal delivery.

**Table 2:** Percentage Distribution on Child's Vital Information

Variables	Frequency (N=120)	Percentage (%)
**Child Age**		
<6 months	73	60.8
6 months – <12 months	33	27.5
12 – 24 months	14	11.7

**Child Birth Position**		
First	34	28.3
Second	42	35.0
Third	33	27.5
Fourth	9	7.5
Fifth and above	2	1.7

**Child Sex**		
Male	63	52.5
Female	57	47.5

**Nature/Type of Delivery**		
Normal delivery	101	84.2
Surgical operation	17	14.2
Traditional birth attendant	2	1.6

### Respondents Infant Feeding Practice

The proportion of respondents that gave their infants' breast milk only in the first 24 hours and 72 hours of birth were 90.8% and 80.8% respectively. About 57.5% of the respondents reported giving their infants breast milk on demand, while 42.5% reported feeding when necessary. Almost (94.2%) all the respondents reported feeding their infants with colostrums. All (100%) respondents reported that their infants respond well to breastfeeding and that they are satisfied with their child's feeding practice. About 55.0% reported giving their infants expressed breast milk if stay away for a long period, as showed in (Table 3).

**Table 3:** Respondents Current Infant Feeding Practice by Frequency and Percentage Distribution

Infant Feeding Practice	Frequency (N=120)	Percentage (%)
**Food received by respondents index child in the first** **24 hours of birth**		
Breast Milk only	109	90.8
Water only	5	4.2
Water with glucose	3	2.5
Nothing	3	2.5

**Food received by respondents index in the first 72hrs** **of birth**		
Breast milk only	97	80.8
Breast milk with water	19	15.8
Baby formula (S.M.A etc)	4	3.3

**How often do you feed your child with breast milk**		
On demand	69	57.5
When necessary	51	42.5

**Did you feed this child with colostrum (first milk)?**		
Yes	113	94.2
No	7	5.8

**Is your child responding well to the feeding?**		
Yes	120	100
No	0	0

**Are you satisfied with your child feeding practice?**		
Yes	120	100
No	0	0

**If you have to stay away from your baby for a long** **period. What food will you make available for the** **baby?**		
Formula food	48	40.0
Expressed breast milk	66	55.0
Water only	6	5.0

### Current Practice Level of Exclusive Breastfeeding among Respondents

**Figure 1:**
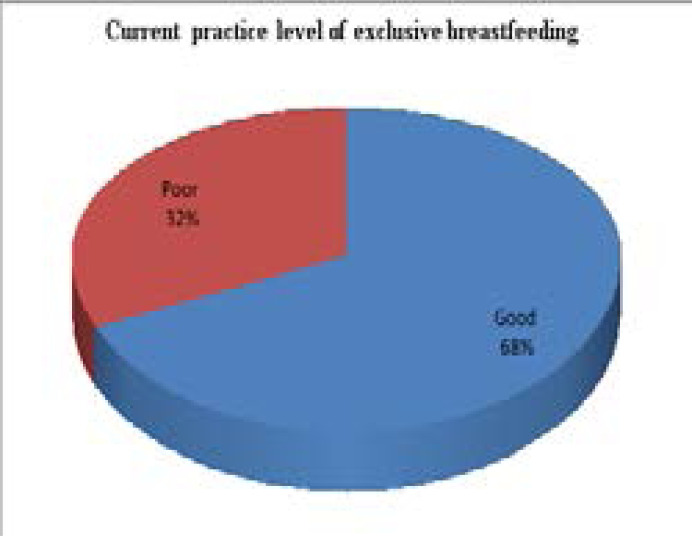
Percentage distribution of current practice level of exclusive breastfeeding

Figure 1 showed that 68.3% of the respondents had good current exclusive breastfeeding practice level.

### Relationship between Demographic Characteristics of Respondents & Respondents' Index Child with Exclusive Breastfeeding Practice

This section shows the bivariate analysis of socio-demographic characteristics of respondents and respondents' index child with exclusive breastfeeding (EBF) practice level. As shown in table 4, the following socio-demographic variables vis marital status, type of marriage, educational status and number of living children were statistically significant with exclusive breastfeeding. Among the various education groups, nursing mothers with postgraduate education had the highest proportion (77.8%) of good EBF practice level compared to others (p=0.02).

**Table 4:** Relationship between Respondents Socio-Demographic Variables & Respondents' Index Child with Exclusive Breastfeeding Practice

Variables	Exclusive Breastfeeding Practice level		Total	Chi-square (χ^2^)	p-value
	Good	Poor			
**Age group (years)**					
≤29	42(67.7)	20(32.3)	62(100)	3.12	0.07
30 – 34	29(74.4)	10(25.6)	39(100)		
≥35	11(57.9)	8(42.1)	19(100)		

**Marital Status**					
Married	81(68.1)	38(31.9)	119(100)	17.85	0.00
Divorced/Widow/Separated/Co-habiting	1(100)	0(0)	1(100)		

**Types of Marriage**					
Monogamous	75(67.6)	36(32.4)	111(100)	12.46	0.00
Polygamous	7(77.8)	2(22.2)	9(100)		

**Religion**					
Christianity	67(69.1)	30(30.9)	97(100)	1.98	0.23
Islam	14(63.6)	8(36.4)	22(100)		
Traditional worshipper	1(100)	0(0)	1(100)		

**Ethnicity**					
Yoruba	45(71.4)	18(28.6)	63(100)	2.96	0.12
Igbo	19(65.5)	10(34.5)	29(100)		
Hausa	4(66.7)	2(33.3)	6(100)		
Others	14(63.6)	8(36.4)	22(100)		

**Educational Status**					
Primary	3(75)	1(25)	4(100)	6.05	0.02
Secondary	28(63.6)	16(36.4)	44(100)		
Tertiary	44(69.8)	19(30.2)	63(100)		
Postgraduate	7(77.8)	2(22.2)	9(100)		

**Residence**					
Rural	9(75.0)	3(25.0)	12(100)	0.08	0.79
Urban	73(67.6)	35(32.4)	108(100)		

**Occupation**					
Full-time house wife	12(75.0)	4(25.0)	16(100)	2.93	0.75
Trader	30(68.2)	14(31.8)	44(100)		
Artisan	11(57.9)	8(42.1)	19(100)		
Civil servant	29(70.7)	12(29.3)	41(100)		

**Number of living children**					
1	25(67.6)	12(32.4)	37(100)	4.94	0.03
2	27(71.1)	11(28.9)	38(100)		
3	21(67.7)	10(32.3)	31(100)		
≥4	9(64.3)	5(35.7)	14(100)		

**Child Age**					
<6 months	53(72.6)	20(27.4)	73(100)	12.65	0.00
6 months – < 12 months	20(60.6)	13(39.4)	33(100)		
12 – 24 months	9(64.3)	5(35.7)	14(100)		

**Child Birth Position**					
First	25(73.5)	9(26.8)	34(100)	0.03	0.43
Second	28(66.7)	14(33.3)	42(100)		
Third	20(60.6)	13(39.4)	33(100)		
Fourth	7(77.8)	2(22.2)	9(100)		
Fifth and above	2(100)	0(0)	2(100)		

**Child Sex**					
Male	43(68.3)	20(31.7)	63(100)	0.97	0.61
Female	39(68.4)	18(31.2)	57(100)		

**Nature/Type of Delivery**					
Normal delivery	69(68.3)	32(31.7)	101(100)	10.42	0.00
Surgical operation	11(64.7)	6(35.3)	17(100)		
Traditional birth attendant	2(100)	0(0)	2(100)		

* Significant at p<0.05

Table 4 also depict that there was significant relationship between age of the index child, nature/type of delivery and EBF practice level. A higher proportion (72.6%) of respondents with infants below six months of age had good EBF practice than those with babies greater than six months of age (p=0.00).

### Logistic Regression Model for Exclusive Breastfeeding (EBF) Practice

The main significant predictors of EBF practice were age of index child, marriage type, educational status of respondents, marital status, and nature/type of delivery (Table 6). Respondents whose child index was less than 6months (<6months) were 5 folds incline to practice good EBF vis-a-vis those whose child were 1 – 2years (OR=5.02, 95% CI=1.28 – 15.43). Those in a monogamous marriage were 3 folds probable to practice good EBF than those in a polygamous setting (OR=3.0, 95% CI=1.80 – 6.73). Respondents who had postgraduate and tertiary education were approximately 4 times and 3 times respectively more likely to have good EBF practice compared with primary school leavers (OR=3.99, 95% CI=1.15 – 9.07; OR=3.12, 95% CI=1.39 – 8.96) (Table 5).

**Table 5:** Logistic Regression Model for Exclusive Breastfeeding Practice

Characteristics	Adjusted Odds Ratio	95% Confidence Interval		p-value
		Lower	Upper	
**Age of child**				
<6 months	5.02	1.28	15.43	0.001*
6 months – < 12 months	1.90	1.34	6.32	0.03*
12 - 24 months (ref)				

**Types of Marriage**				
Monogamous	3.0	1.80	6.73	0.004*
Polygamous (ref)				

**Educational Status**				
Postgraduate	3.99	1.15	9.07	0.001*
Tertiary	3.12	1.39	8.96	0.002*
Secondary	1.98	1.04	3.72	0.01*
Primary (ref)				

**Marital Status**				
Married	2.0	0.1	0.8	0.01*
Divorced/Widow/Separated/Co- habiting (ref)				

**Number of living children**				
1	3.4	0.8	7.2	0.06
2	0.7	0.4	1.3	0.45
3	1.9	0.7	7.4	0.13
≥4(ref)				

**Nature/Type of Delivery**				
Normal delivery	2.96	1.75	7.48	0.004*
Traditional birth attendant	1.04	0.57	4.03	0.212
Surgical operation(ref)				

(Ref) Reference category

* Significant at p<0.05

Respondents who were married are twice more likely to have good EBF practice than those that are single (OR=2.0, 95% CI=0.1 – 0.8). Similarly, respondents who had their children through normal delivery are more likely have good EBF practice level than those that had their children through surgical operation (OR=2.96, 95% CI=1.75 – 7.48) as depicted in Table 5.

It is important to note that the wide confidence interval observed for some of the dependent variables in Table 5 is due to the small sample size used in the study.

## Discussion

It has been established that EBF of infants for the first semester of life is the ideal requirement for babies and juveniles to have wholesome growth and well-being^7^. Likewise studies have shown that mothers who practice EBF are wholesome, capable of even child spacing, developed strong psychosocial bond with their progenies and have lower incidence of breast cancer vis-a-vis their counterparts who do not practice EBF^10^, ^25^–^27^.

In the present study, Early Initiation of Breastfeeding (EIBF) was reported to be 90.8%. This increased tremendously compared to the 24.9% EIBF reported by the National Demographic and Health Survey (NDHS) in 2013^28^ following the introduction of the BFHI, National Breastfeeding Policy and the National Policy on IYCF in 1992, 1998 and 2005 respectively. In consonance with this finding, several studies also reported majority of mothers initiating early breastfeeding: Lagos^29^, Sokoto^26^, Osun^9^, and Anambra^30^ as recommended by WHO and UNICEF^2^. Based on the report of Adhikari^31^, approximately 50% of the respondents conceived the opinion that infants feeding be based on demands; likewise, this study reported that a greater proportion 57.5% of the respondents have erudition that infants breastfeeding arise from demand. The practice of feeding child with colostrum was reported by almost 94.2% all the mothers. This practice contrast what was reported by Ajibuah^32^ in his study, where the practice of abrogating colostrum and substituting it with diverse forms of prelacteal feeds was noticed in more than half of the communities in Yola state.

According to the 2014 NDHS survey, the prevalence of EBF rate in Nigeria is estimated at 17%^28^, but the current EBF rate of 68.3% reported in this survey was significantly greater vis-à-vis the national rate and that reported by other Nigerian studies^32^–^34^, ^26^. The rate of practice of EBF obtained in the study was high and similar to the percentage reported beforehand by other authors^35^–^36^. According to Agunbiade and Ogunleye^9^, some nursing mothers in western Nigeria practice EBF based on the erudition that it aids neophytes to grow wholesomely. The high rate of good current EBF practice level observed in this study may be attributed to the awareness created by the implementation of various policies and guidelines on IYCF by the Federal Government of Nigeria. In comparison to other third world nations vis India, Indonesia and Ethiopia where the national policy, strategic action plans and health system framework to aid IYCF practices are weak. Nigeria has established a national legislative and health system framework to enhance infant and young child feeding practices^37^. In spite of all these initiatives, malnutrition, and under-five children feeding related ailments and mortality still persist as a public health issue in the country^38^. Moreover, there has been a drastic reduction in the proportion of children under 24 months of age who were fed in consonance with IYCF (breastfeeding and complementary feeding) guidelines in Nigeria, from 30% in 2009^14^ to 10% in 2014^28^.

Although exclusive breastfeeding is associated with numerous rewards which suffice as stimulation for mothers to practice it, albeit, several mothers were defiant or apathetic to exclusively breastfeed their wards for diverse motives^7^. Multiple logistic regressions revealed that the main significant predictors of EBF practice in this study were age of index child, types of marriage, educational status of respondents, marital status, and nature/type of delivery. In this study, mothers from monogamous setting are three (3) times more probable to practice EBF than those from polygamous setting. Hence, this study revealed that the type of marriage have a strong impacts on mother's decision to exclusively breastfed. This may be due to the fact that nursing mothers from polygamy are faced with more economic challenges than their counterparts from monogamous setting. Several studies have reported that women from higher socio-economic class (HSES) are more dispose to practice EBF vis-à-vis those from the lower socio-economic class (LSES)^39^–^40^. Conversely, in Nigeria, it is consequential to note that mothers from HSES group show more inclination towards bottle feeding compared to mothers from LSES because they are more likely to be employed, are more accessible to advertising gadgets and are more financially empowered to purchase breast milk substitutes^41^. The violation of the International Code of Marketing of Breastmilk Substitutes (the codes) has been reported in numerous countries, including Nigeria^42^–^44^. Major reasons enunciated for bottle feeding infants by HSES group include poor policy implementation of the Code and herculean working conditions that are not favourable to breastfeeding mothers^41^,^45^.

The impact of education on infant feeding practices differs from one location to other^46^. This study showed that women with secondary and post-secondary education are more probable to practice EBF compare with primary school leaver. Failure to practice EBF and prelacteal feeding has been attributed to low level of maternal education^19^. Conversely, other study attributed poor educational status of mothers to elevated breastfeeding practices^17^. Erudite women have the potential to practice EBF than their illiterate counterparts (who only breastfeed longer based on tradition) since they have better knowledge of the benefits associated with breastfeeding. An enlightenment program in diverse language that emphasized the importance of EBF could be implemented to promote the practice. The marital status of a woman has been identified as a vital parameter of baby feeding patterns in many societies^46^–^47^. Regression analysis from this study revealed that married women are twice more likely to practice EBF than single/divorced/separated mothers. Previous authors have reported high prevalence of suboptimal infant feeding among single mothers^48^–^49^. Studies have established that single mothers are less probable to breastfeed longer and adequately vis-à-vis their married counterparts who received psychological and emotional support from their partners^50^–^52^.

This study showed that respondents who had vaginal delivery are three (3) folds more probable to practice EBF vis-à-vis those that had their progeny through surgical operation. Mothers who had their children via caesarean section tend to displayed negative disposition to breastfeeding and had strenuous encounters with infant breastfeeding compared with women who had vaginal delivery^53^–^54^. The WHO/UNICEF introduced the BFHI to support and aid nursing mothers at health facilities. Nevertheless, majority of women in many poor economy countries deliver their progenies at home^55^, which poses a huge impediment to the actualization of the goals of BFHI. In Nigeria, most mother (∼64%) deliver their babies at home, aided by traditional birth attendant or a family member, who are often not certified to provide appropriate infant feeding options to new mothers^56^,^28^. An assessment of the BFHI status in Nigeria found that only an insignificant proportion of hospitals, 8% as reported by UNICEF^37^ and 95 out of 25,000 hospitals, or 0.004% as enunciated by Nigerian Ministry of Health^45^ were BFHI certified. This vital component of the BFHI has practically “failed”.

Nigeria has a well-defined IYCF legislation, community based strategies and communication, and health system level actions, including suitable monitoring and evaluation programmes to enhance IYCF practices^37^. In spite of this, political apathy and moribund subnational government committees to fully implement vital IYCF policies, such as the BFHI, National Breastfeeding Policy, National IYCF policies among others, are likely reasons for Nigeria's poor IYCF practices^45^. Furthermore, socio-economic factors (poor household empowerment and low maternal education), individual factors (younger maternal and child age), and health service factors (fewer antenatal care visits and home delivery) are also contributory factor to the low IYCF practices.

## Limitations of this study

In spite of the high response rate and importance of exclusive breastfeeding to the survival of new born and infant children, there are vital limitations to this survey. Firstly, some of the respondents shown uncooperative attitude and had negative attitude towards the study due to socio-cultural reasons. Secondly, thirteen (13) questionnaires were not properly or incomplete filled, rendering them less irrelevant. Thirdly, the study was limited to a health facility in one LCDA in Alimosho LGA; therefore results may not be generalisable to all nursing mothers in Lagos State or the country at large. Fourthly, this survey was solely quantitatively with no opportunity for respondents to indite their comments or express their perspective outside the questions asked. Fifthly, the study fails to collect qualitative information on the sociocultural beliefs and practices on breastfeeding. Hence, future research should include a qualitative methodology to aggrandise findings in this area. Lastly, the use of cross-sectional design in the study only provides a snap shot of the situation and does not provide a casual inference.

## Way forward

Sustainable Development Goals (SDG) 2 and 3, advocate for improved nutrition, and salubrious lives for all^57^. Healthy lifestyle begins with higher uptake of breastfeeding and nutritionally endowed foods at weaning. To achieve these and other IYCF policies, the Nigerian government need to design and implement context specific initiatives and policies that efficiently corroborates the healthcare system; aids optimal infant and juvenile feeding practices; appropriate regulation of baby foods market; initiation of community based programmes that involve family members in health information sessions, and tailored towards the specific socio-cultural context in which the woman live; and enacting mother-friendly workplace legislation. Improvements in the IYCF practices would consequently lead to the attainment of the Global Nutrition Target by 2025, where Global Target 5 is aimed at increasing exclusive breastfeeding rate in the first six months up to at least 50% by the year 2025^58^.

Also, the implications of the impact, or otherwise, of national policies on trends in IYCF practices is that there is an urgent needs for the government to adequately and consistently fund the health care sector in line with the commitment made by the African Union leaders, including Nigeria, to appropriate minimum of 15% of their annual budget to improve the health sector. Health care spending in the Nigeria context must be transparent, monitored and evaluated periodically given the mismanagement of previous fund^59^,^44^.

## Conclusions

The objective of this survey was to explore the predictors of exclusive breastfeeding (EBF) practice among nursing mothers attending the IsheriOlofin Primary Health Centre in Lagos State, Nigeria. The study revealed that the main predictors of EBF practice among nursing mothers were: age of index child, marriage type, maternal educational level, marital status, and nature/type of delivery. Although, respondents in this study demonstrated high rate and good current exclusive breastfeeding practice level, this however does not translate to a better national IYCF practices.

Hence, this study recommends that health education principles and community based programmes be implemented to tackle the socio-economic and demographic challenges identified in this study. Also, the government as a matter of urgency should strengthen community and facility based participation, and integrated IYCF policy implementations. Conclusively, it is expedient that research be conducted to inquire into the merits of EBF practice in sub-Saharan Africa. Such studies should also delve into the intrinsic factors that serve as barriers to EBF practice among nursing mothers.

## References

[1] Black RE, Allen LH, Bhutta ZA, Caulfield LE, de Onis M, Ezzati M (2008). Maternal and child under-nutrition: global and regional exposures and health consequences. Lancet.

[2] World Health Organization (2001). Global Strategy for infant and young child feeding: The optimal duration of exclusive breastfeeding. Fifty World Health Assembly.

[3] World Health Organization (2009). Infant and young child feeding (IYCF). Model Chapter for textbooks for medical students and allied health professionals.

[4] UNICEF, United Nations Children's Fund (2018). WHO and UNICEF issue new guidance to promote breastfeeding in health facilities globally.

[5] WHO, World Health Organisation (2009). Infant and young child feeding (IYCF). Model Chapter for textbooks for medical students and allied health professionals.

[6] UNICEF, United Nations Children's Fund (2013). Breastfeeding.

[7] Sokan-Adeaga MA, Sokan-Adeaga AA, Sokan-Adeaga ED (2019). “A Systematic Review on Exclusive Breastfeeding Practice in Sub-Saharan Africa: Facilitators and Barriers”. Acta Scientific Medical Sciences.

[8] National Population Comission, NPC/Nigeria and ICF (2019). Nigeria Demorgraphic and Health Survey 2018.

[9] Agunbiade OM, Ogunleye OV (2012). Constraints to exclusive breastfeeding practice among breastfeeding mothers in Southwest Nigeria: implications for scaling up. Int Breastfeed J.

[10] Aborigo RA, Moyer CA, Rominski S, Adongo P, Williams J, Logonia G (2012). Infant nutrition in the first seven days of life in rural northern Ghana. BMC Pregnancy Childbirth.

[11] Kakute PN, Ngum J, Mitchell P, Kroll KA, Forgwei GW, Ngwang LK, Meyer DJ (2005). Cultural barriers to exclusive breastfeeding by mothers in a rural area of Cameroon, Africa”. J of Midwifery & Women's Health.

[12] Central Statistical Agency [Ethiopia] and ORC Macro (2006). Ethiopia Demographic and Health Survey (EDHS) 2005.

[13] Federal Ministry of Health (2005). National Strategy for Child Survival in Ethiopi.

[14] Alemayehu T, Haidar J, Habte D (2009). Determinants of exclusive breastfeeding practices in Ethiopia. Ethiop J Health Dev.

[15] Aryeetey ONR, Antwi LC (2013). “Re-assessment of selected Baby-Friendly maternity facilities in Accra, Ghana”. Int Breastfeed J.

[16] Otoo GE, Lartey AA, Pérez-Escamilla R (2009). Perceived incentives and barriers to exclusive breastfeeding among peri-urban Ghanaian women. J of human lactation.

[17] Lawoyin TO, Olawuyi JF, Onadeko MO (2001). Factors associated with exclusive breastfeeding in Ibadan, Nigeria. J of Human Lactation: Official Journal of International Lactation Consultant Association.

[18] Qureshi AM, Oche OM, Sadiq UA, Kabiru S (2011). Using community volunteers to promote exclusive breastfeeding in Sokoto state, Nigeria”. Pan Afri Med J.

[19] Ogunlesi TA (2010). “Maternal socio-demographic factors influencing the initiation and exclusivity of breastfeeding in a Nigerian semi-urban setting”. Maternal and Child Health Journal.

[20] Issaka AI, Agho KE, Page AN, Burns P, Stevens GJ, Dibley MJ (2014). Determinants of early introduction of solid, semi-solid or soft foods among infants aged 3–5 months in four Anglophone West African Countries”. Nutrients.

[21] Arora S, McJunkin C, Wehrer J, Kuhn P (2000). Major factors influencing breastfeeding rates. Mother's perception of father's attitude and milk supply. Pediatrics.

[22] Salami LI (2006). “Factors influencing breastfeeding practices in Edo state, Nigeria”. African Journal of Food, Agriculture, Nutrition and Development.

[23] Roudbari M, Roudbari S, Fazaeli A (2009). Factors associated with breastfeeding patterns in women who recourse to health centres in Zahedan, Iran. Singapore Med J.

[24] Lagos State Government (2013). Abstract of Local Government Statistics.

[25] Kramer MS, Kakuma R (2002). “The optimal duration of Exclusive breastfeeding. A systematic review”.

[26] Oche MO, Umar AS, Ahmed H (2011). Knowledge and practice of exclusive breastfeeding in Kware, Nigeria. Afr Health Sci.

[27] United Nations Children's Fund (UNICEF) (1999). Breastfeeding: Foundation for a healthy future.

[28] National Population Commission (NPC) [Nigeria] and ICF International (2014). Nigeria Demographic and Health Survey 2013.

[29] Adebayo AA, Leshi OO, Sanusi RA (2014). Breastfeeding Knowledge and Practice of Mothers with Infants less than Six Months Old, in Kosofe Local Government of Lagos State. Niger J of Nutritional Sci.

[30] Ukegbu AU, Ebenebe EU, Ukegbu PO, Onyeonoro UU (2011). Determinants of breastfeeding pattern among nursing mothers in Anambra State, Niger. East Afr J Public Health.

[31] Adhikari TM (2014). Knowledge and Practice of Mother regarding Exclusive Breastfeeding Having Infant at a Tertiary Level Hospital, Kathmandu. J Nepal Paediatr Soc.

[32] Ajibuah BJ (2013). Appraisal of nursing mothers' knowledge and practice of exclusive breastfeeding in Yobe state, Nigeria. J BiolAgric Healthcare.

[33] Iliyasu Z, Kabir M, Abubakar IS, Galadanci NA (2005). Current knowledge and practice of exclusive breastfeeding among mothers in Gwale Local Government Area of Kano State. Niger Med Practitioner.

[34] Essien NC, Samson-Akpan PE, Ndebbio TJ, John ME (2009). Mothers' knowledge, attitude, belief and practices concerning exclusive breastfeeding in Calabar, Nigeria. Africa J Nurs Midwifery.

[35] Ukegbu AU, Ebenebe EU, Ukegbu PO (2010). Breastfeeding pattern, anthropometry and health status of infants attending child welfare clinics of a teaching hospital in Niger. S Afr J Clin Nutr.

[36] Sanusi RA, Leshi OO, Agada VN (2016). Mother's Knowledge and practice of breastfeeding and complimentary feeding in Enugu state, Nigeria. Journal of Research in Nursing and Midwifery (JRNM) (ISSN: 2315 – 568).

[37] United Nation Children's Education Fund (2012). Infant and young child feeding programming status: results of 2010 - 2011 assessment of key actions for comprehensive infant and young child feeding programmes in 65 countries. Nutrition section.

[38] United Nation Children's Fund – Nigeria (2016). Maternal and Child health.

[39] Agho KE, Dibley MJ, Odiase JI, Ogbonmwan SM (2011). Determinants of exclusive breastfeeding in Nigeria. BMC Pregnancy Childbirth.

[40] Velpuri J (2004). Breastfeeding Knowledge, and Attitudes, Beliefs, and Intentions regarding Breastfeeding in the Workplace among Students and Professionals in Health Related Fields.

[41] Ogbo FA, Page A, Agho KE, Claudio F (2015). Determinants of trends in breast-feeding indicators in Nigeria, 1999–2013. Public Health Nutr.

[42] Piwoz EG, Huffman SL (2015). The impact of marketing of breast-milk substitutes on WHO- recommended breastfeeding practices. Food Nutr Bull.

[43] Taylor A (1998). Violation of the international code of marketing of breast milk substitutes. Prevalence in four countries. BMJ.

[44] Ogbo FA, Page A, Idoko J, Claudio F, Agho KE (2016). Have policy responses in Nigeria resulted in improvements in infant and young child feeding practices in Nigeria?. Int Breastfeed J.

[45] Ngozi N, Nte A, Federal Ministry of Health – Nigeria (2015). The World Breastfeeding Trends Initiative (WBTi) – Nigeria.

[46] Ajibade B, Okunlade J, Makinde O, Amoo P, Adeyemo M (2013). Factors influencing the practice of exclusive breastfeeding in rural communities of Osun state, Nigeria. European Journal of Business and Management.

[47] Victor R, Baines SK, Agho KE, Dibley MJ (2013). Determinants of breastfeeding indicators among children less than 24 months of age in Tanzania: a secondary analysis of the 2010 Tanzania demographic and health survey. BMJ Open.

[48] Kimani-Murage EW, Madise NJ, Fotso JC, Kyobutungi C, Mutua MK, Gitau TM (2011). Patterns and determinants of breastfeeding and complementary feeding practices in urban informal settlements, Nairobi Kenya. BMC Public Health.

[49] Tampah-Naah AM, Kumi-Kyereme (2013). Determinants of exclusive breastfeeding among mothers in Ghana: a cross-sectional study. International Breastfeeding Journal.

[50] Okafor AE, Agwu PC, Okoye UO, Uche OA, Oyeoku EK (2018). Factors associated with exclusive breastfeeding practice among nursing mothers in rural areas of Enugu state and its implications for social work practice in Nigeria. Social Work in Public Health.

[51] Onah S, Osuorah DIC, Ebenebe J, Ezechukwu C, Ekwochi U, Ndukwu I (2014). Infant feeding practices and maternal socio-demographic factors that influence practice of exclusive breastfeeding among mothers in Nnewi South-East Nigeria: A cross-sectional and analytical study. International Breastfeeding Journal.

[52] Hunegnaw MT, Gezie LD, Teferra AS (2017). Exclusive breastfeeding and associated factors among mothers in Garmin District, northwest Ethiopia: A community based cross-sectional study. International Breastfeeding Journal.

[53] Imhonde H, Shaibu H, Imhonde J, Handayani L (2012). Type of Birth, Depression and Anxiety as determinates of Breastfeeding Attitude among Nursing Mothers. International Journal of Public Health Science (IJPHS).

[54] Carlender AK, Edman G, Christensson K, Andolf E, Wiklund I (2010). Contact between mother child and partner and attitudes towards breastfeeding in relation to mode of delivery. Sexual and Reproductive Health Care.

[55] Montagu D, Yamey G, Visconti A, Harding A, Yoong J (2011). Where do poor women in developing countries give birth? a multi-country analysis of demographic and health survey data. PLoS One.

[56] Ogbo FA, Agho KE, Page A (2015). Determinants of suboptimal breastfeeding practices in Nigeria: evidence from 2008 demographic and health survey. BMC Public Health.

[57] Madeley J (2015). Sustainable development goals. Appropriate Technol.

[58] World Health Organisation (2014). Global nutrition targets 2025: policy brief series (WHO/NMH/NHD/14.2).

[59] Daniel S (2015). We don't need foreign aid – Buhari. In: The Vanguard.

